# The over-expression of survivin enhances the chemotherapeutic efficacy of YM155 in human hepatocellular carcinoma

**DOI:** 10.18632/oncotarget.3337

**Published:** 2015-01-21

**Authors:** Hongping Xia, Jianxiang Chen, Ming Shi, Amudha Deivasigamani, London Lucien P.J. Ooi, Kam M. Hui

**Affiliations:** ^1^ Laboratory of Cancer Genomics, Division of Cellular and Molecular Research, Humphrey Oei Institute of Cancer Research, National Cancer Centre, Singapore; ^2^ Department of Hepatobiliary Oncology, Cancer Center, Sun Yat-sen University, Guangzhou, P.R. China; ^3^ Department of Surgical Oncology, National Cancer Centre, Singapore; ^4^ Department of General Surgery, Singapore General Hospital, Singapore; ^5^ Cancer and Stem Cell Biology Program, Duke-NUS Graduate Medical School, Singapore; ^6^ Department of Biochemistry, Yong Loo Lin School of Medicine, National University of Singapore, Singapore; ^7^ Institute of Molecular and Cell Biology, A*STAR, Biopolis Drive Proteos, Singapore

**Keywords:** Cancer heterogeneity, DNA damage, Hepatocellular carcinoma, Survivin, YM155

## Abstract

Hepatocellular carcinoma (HCC) is the second leading cause of cancer-related deaths worldwide. The inability of chemotherapeutic drugs to selectively target HCC tumor cells because of their predominant resistant phenotype to most conventional anticancer agents bestows a major obstacle for the clinical management of HCC. In this report, we have examined and demonstrated the remarkable heterogeneity of expression of survivin and its phosphorylated active form (p-survivin) in HCC patients' tissues and cell lines. Furthermore, the expression of survivin and p-survivin in HCC cell lines was found to be associated with response to the small-molecule survivin suppressant YM155. Therefore, in the HCC cell lines that express elevated level of survivin and p-survivin, YM155 efficiently inhibited their proliferation, induced cell cycle arrest and apoptosis resulting in DNA damage through the dysregulation of cell-cycle checkpoint-related regulatory genes. Importantly, YM155 yielded significantly better therapeutic effect than sorafenib when tested in an orthotopic mouse model using patient-derived HCC xenografts with elevated survivin and p-survivin expression. Our results clearly demonstrated that the level of survivin and p-survivin expression could serve as molecular predictive biomarkers to select potential YM155-responsive patients, in a move towards delivering precision medicine for HCC patients.

## INTRODUCTION

Hepatocellular carcinoma (HCC) is the second leading cause of cancer-related deaths worldwide [1]. Unfortunately, most HCCs are still being diagnosed at their late stages due to the lack of efficient early detection and surveillance strategies [2, 3]. Despite novel molecularly targeted therapies [4] and immunotherapy [5] are being developed, they are not very effective against advanced HCC. Currently, sorafenib is the only FDA-approved molecular inhibitor for the systemic therapy of advanced HCC. Although data from the Sorafenib Hepatocellular Carcinoma Assessment Randomised Protocol (SHARP) trial [6] and the Asia-Pacific study [7] could demonstrate a significant survival benefit, the absolute gain in life expectancy was marginal (10.7 versus 7.9 months with placebo). Despite many ongoing efforts to explore non-surgical strategies to treat HCC, they have not been very successful and over 80% of the phase 3 molecularly-related targeted trials have failed [8]. Hence, systemic chemotherapy for advanced HCC remains an urgent unmet medical need. One possible reason was the lack of companion biomarkers that can identify patient subpopulations that are potentially responsive to the treatment in many of these studies [9]. Recent advances in oncology to identify inter-patient heterogeneity enable the stratification of patients for targeted treatments [10]. Although the cause of genetic heterogeneity in cancer has not been well-established, there is the strategy approach to identify prognostic biomarkers and to decipher the associated molecular mechanisms to facilitate the selection of patients for molecularly-targeted therapies [11].

Survivin (BIRC5) is a member of the inhibitor-of-apoptosis proteins (IAPs) family that is overexpressed in most of human tumors but not in normal tissues [12]. YM155, a survivin suppressant, has been tested as a single agent to treat patients with melanoma, lymphoma, lung and prostate cancer [12]. In several phase I/II clinical trials, YM155 has been shown to be well tolerated, but unfortunately, its clinical efficacy has been insofar only moderate [13]. One possible reason may be the lack of information on the molecular heterogeneity of the tumors being treated and consequently, making it difficult to interpret the overall biological response. Over-expression of survivin in HCC has been reported to correlate with poor prognosis [14]. In this study, we hypothesized that targeting survivin could be a treatment strategy for HCC and subsequently demonstrated the remarkable heterogeneity of expression of survivin and its phosphorylated active form (p-survivin) in HCC patients' tissues and cell lines. High survivin and p-survivin expression in HCC cells associated with increased therapeutic response to YM155.

## RESULTS

### The remarkable heterogeneous survivin expression in HCC clinical samples

**Figure 1:**
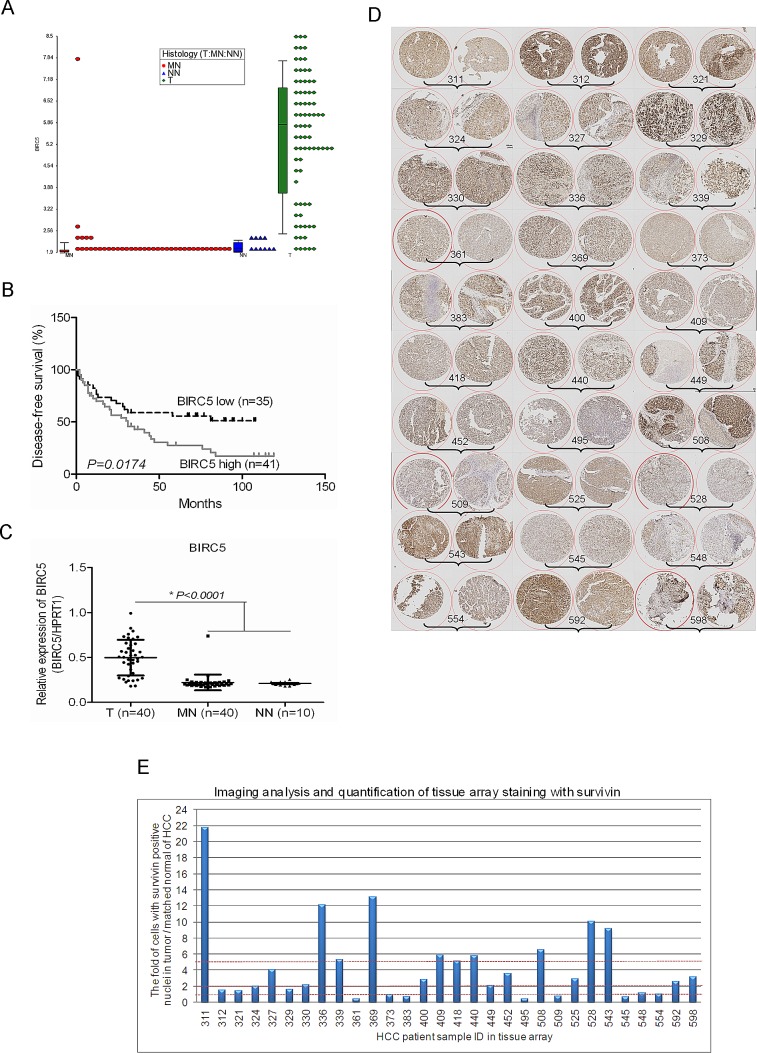
The remarkable heterogeneous expression of survivin in HCC tissues (A) Expression of BIRC5 was shown by dot plot analysis, by searching the HCC Gene Expression database established in our laboratory using Affymetrix Human Genome U133 plus 2.0 Arrays (Affymetrix, Santa Clara, CA, USA) comprising of HCC tumour and adjacent histologically normal liver tissues (T: Tumor, MN: Matched Normal, NN: Normal Normal (histologically normal liver tissues of colorectal cancer patients)). (B) Expression of BIRC5 was associated with the disease-free survival of patients with HCC. The median expression value obtained for BIRC5 of the samples was chosen as the cut-off point for survival analysis using the Kaplan-Meier method. (C) Validation of the expression of BIRC5 in another 40 pairs of HCC tumour and matched normal tissue samples as well as 10 cases of normal liver tissues by RT-qPCR. (D) The image of tissue array IHC staining for validation the expression of survivin in another panel of HCC tumour tissues. (E) The imaging analysis and quantification of tissue array staining with survivin. The 60 spots in the tissue array slide were from 30 patients' HCC samples and duplicate spots for each patient. The IHC images were evaluated according to the percentage of cells with positive nuclei.

The expression of survivin is quiescent in most normal, terminally differentiated tissues but it is widely expressed in cancers, including HCC. Previously, we have established a global gene expression database on human HCC tumour samples and adjacent histologically normal liver tissues using Affymetrix Human Genome U133 plus 2.0 Arrays [15, 16]. To evaluate the potential role of survivin as a therapeutic target for HCC, we systematically examined the expression of survivin in our dataset. The expression of survivin was found to be remarkably heterogeneous in HCC clinical samples, the detected signal intensity ranged from log_2_2 to log_2_9 (Fig. 1A). Furthermore, when survival analysis was performed using the median expression values of survivin, elevated survivin expression in HCC tissues was significantly associated with shorter disease-free survival, p=0.0174 (Fig. 1B). The relationship between survivin expression and the clinicopathological parameters of the HCC samples studied was shown in the supplementary Table S1. The heterogeneous expression of survivin in HCC clinical samples was also observed in the published dataset of Roessler et al [17] (Fig. S1A).

The heterogeneous expression of survivin was further validated in 40 pairs of HCC samples by real time RT-qPCR analysis (Fig. 1C) and an additional independent cohort of 30 pairs of HCC samples by Immunohistochemistry (IHC) (Fig. 1D, 1E and Fig. S1B). The heterogeneous expression of survivin studied by IHC was quantitated in cells with positive nuclei staining. There were 24 out of 30 (80%) of HCC samples that yielded an elevated expression of survivin. Among these, 18 out of 30 (60%) gave a 2-fold increase in survivin expression while 10 out of 30 (33.33%) gave a 5-fold increase in survivin expression (Fig. 1D and 1E).

### The expression of survivin and its phosphorylated active form (p-survivin) in HCC cell lines correlated with their sensitivity to the survivin suppressant YM155

**Figure 2:**
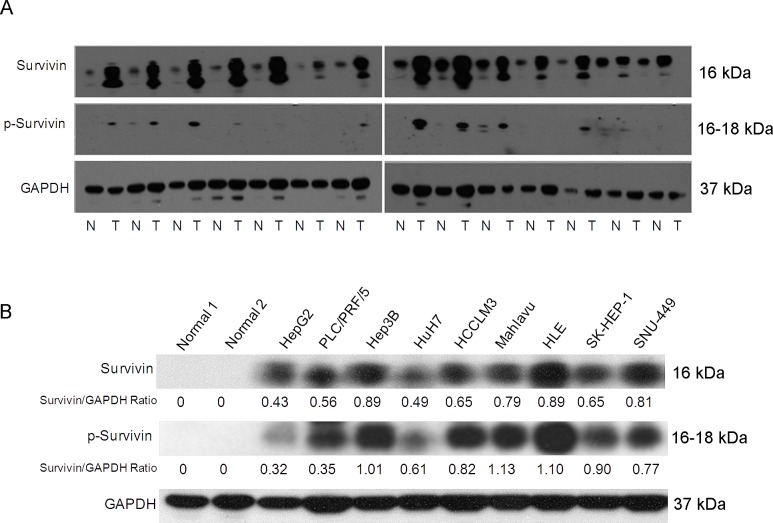
The heterogeneity expression and phosphorylation status of survivin affects sensitivity to the survivin suppressant YM155 in HCC cells (A) The expression and phosphorylation status of survivin protein in a panel of HCC patients' tumor (T) and histologically matched normal (N) tissue samples by Western Blot analysis. (B) The expression and phosphorylation status of survivin protein in a panel of liver cancer cell lines and two normal liver tissues by western blotting analysis.

The observation that survivin expressed in human cancer cells but is absent from most normal adult tissues makes it a promising therapeutic target for cancer chemotherapy. The phosphorylation of survivin at Thr34 is required for its role in regulating the cell cycle. We therefore examined the expression of survivin and p-survivin in a panel of HCC tissues (Fig. 2A) and cell lines (Fig. 2B) by western blotting. Consistent with the microarray data, survivin expression was heterogeneous. We also observed the expression of survivin and p-survivin was high in the cell lines Hep3B, HLE and Mahlavu compared to HepG2 and HuH7 cells (Fig. 2B). However, there are no correlation among the liver cancer cells with different levels of survivin and cell growth rates, invasiveness, and EMT markers (Vimentin and E-cadherin) expression (Fig. S2).

**Figure 3:**
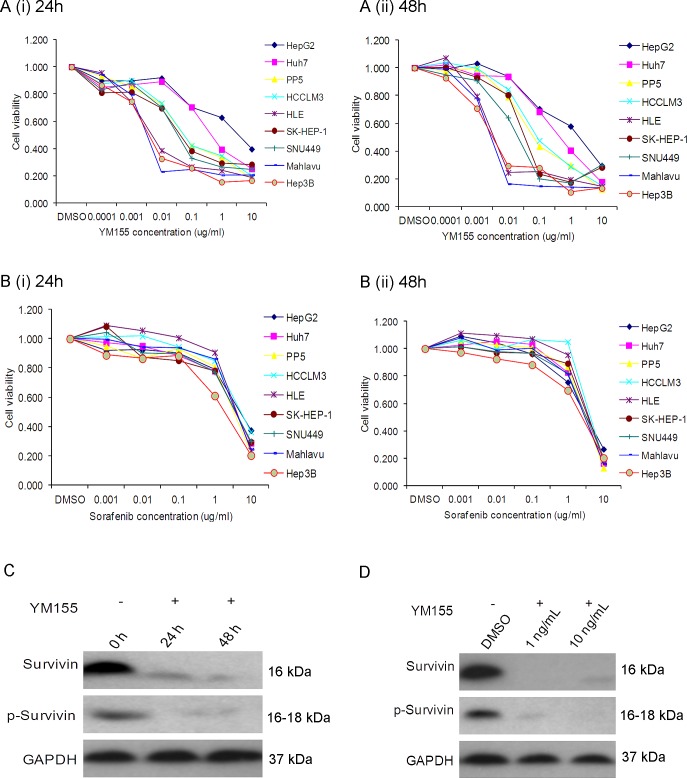
YM155 inhibits cell growth in sensitive liver cancer cells (A) Incubation of HCC cell lines in YM155 for (i) 24h and (ii) 48h at different concentrations; (B) Incubation of HCC cell lines in sorafenib for (i) 24h and (ii) 48h at different concentrations. (C) Western blot analysis of Mahlavu cells after incubating for either 24h or 48h in 10ng/ml (C) and 48h treatment 1 ng/ml or 10 ng/ml of YM155 (D).

Next, we compared the chemosensitivity of these HCC cells to YM155, a survivin inhibitor. It was observed that HCC cells exhibited different sensitivity to YM155 (Fig. 3A). The observed concentration for 50% of maximal effect (EC50) of YM155 for the high survivin-expressing cells such as Mahlavu and HLE was <10ng/ml. In comparison, the observed EC50 of YM155 for the low survivin-expressing cell lines HuH7 and HepG2 was >1000ng/ml after incubating for either 24h or 48h (Fig. 3A (i) and (ii), respectively). In contrast, sorafenib gave indiscriminately an overall EC50 >1000ng/ml for most of the HCC cells tested (Fig. 3B (i) and (ii)). It appeared likely that the sensitivity of HCC cells to YM155 could be associated with their respective level of survivin and p-survivin expression: high survivin-expressing cells such as Mahlavu and HLE are more sensitive to YM155 than the low survivin-expressing HCC cells such as HuH7 and HepG2. This observation was also confirmed by Western blotting: 10ng/ml and 1ng/ml of YM155 significantly inhibited the expression of survivin in the Mahlavu cells after incubating for either 24h or 48h (Fig. 3C and 3D respectively).

### YM155 inhibits the anchorage-independent cell growth, induced cell cycle arrest, apoptosis and DNA damage of high survivin-expressing sensitive HCC cells

**Figure 4:**
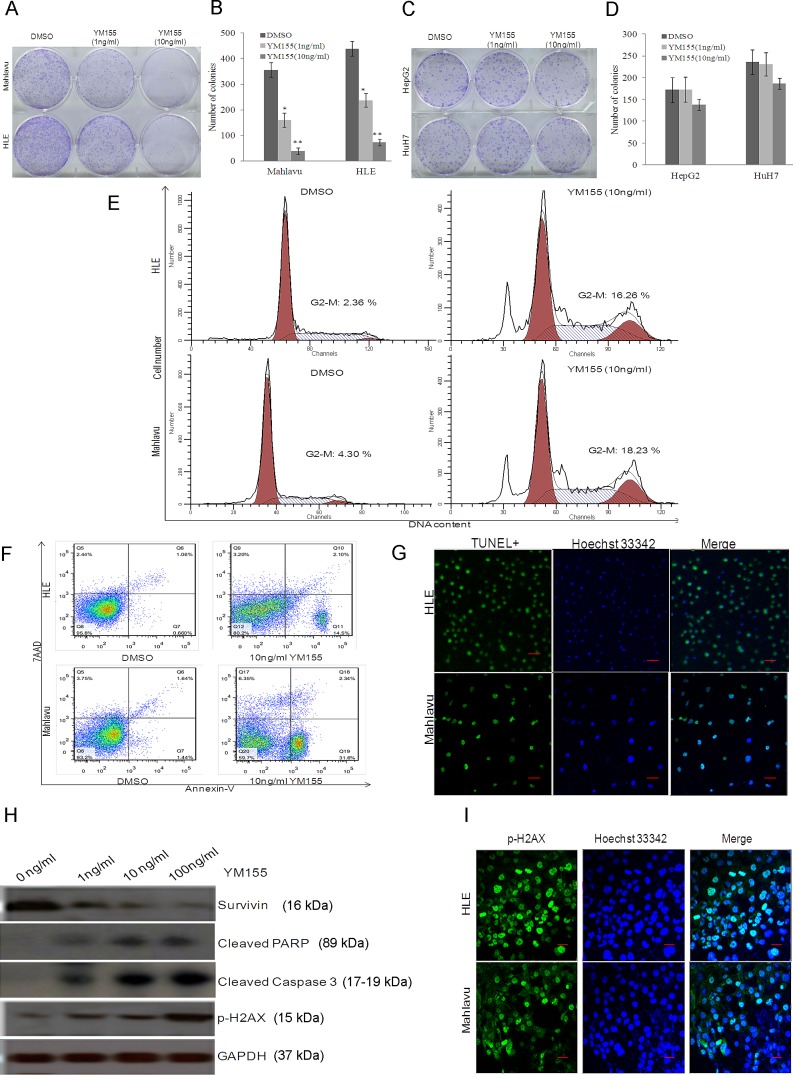
YM155 inhibits the anchorage-independent cell growth, induced cell cycle arrest, apoptosis and DNA damage of high survivin-expressing sensitive HCC cells Representative image (A) and statistical analysis (B) of the inhibition effect of YM155 on anchorage-independent cell growth of sensitive Mahlavu and HLE HCC cells (* P<0.05, ** P<0.01). Representative image (C) and statistical analysis (D) of the inhibition effect of YM155 on anchorage-independent cell growth of resistant HepG2 and HuH7 HCC cells. (E) Representative images of the effect of YM155 on cell cycle progression. The G2/M phase arrest was observed in both Mahlavu and HLE cells treated with YM155. (F) The increase in Annexin-V^+^ cells was observed by flow cytometry analysis in both Mahlavu and HLE cells treated with YM155. (G) The increasing of TUNEL^+^ cells was observed by confocal microscopy analysis in both Mahlavu and HLE cells treated with YM155. (H) Increase in cleaved Caspase 3 and PARP, P-H2AX could be detected by Western blot following treatment of Mahlavu cells with increasing doses of YM155. (I) Mahlavu and HLE cells were treated with 10ng/ml YM155 and stained for P-H2AX and Hoechst 33342 and analysed by confocal microscopy. The green signal represents staining for P-H2AX. Nuclear DNA was detected by staining with Hoechst 33342.

The ability of cancer cells to grow under anchorage-independent condition is a property associated with tumorigenesis. When Mahlavu and HLE cells were plated separately in soft agar and treated with YM155 at 1ng/ml and 10ng/ml, both Mahlavu and HLE cells showed a statistically significant reduction in anchorage-independent growth versus the medium controls (Fig. 4A and 4B). In comparison, treatment of HuH7 and HepG2 cells with YM155 under similar conditions gave insignificant inhibition (Fig. 4C and 4D).

Survivin is a member of the inhibitor of apoptosis (IAP) gene family that plays important roles in regulating cell division and apoptosis. When YM155-sensitive Mahlavu and HLE cells were treated with 10ng/ml YM155 for 12h, significant cell cycle arrest in the G2/M phase was observed. The G2/M phase cell sub-population of the HLE cells increased approximately 7-fold and a 4-fold increase was observed with the Mahlavu cells (Fig. 4E). Moreover, TUNEL-positive cells were significantly increased for the YM155-sensitive Mahlavu and HLE cells (Fig. 4F and 4G) compared to the YM155-resistant HuH7 and HepG2 cells (Fig. S3A) after incubating with 10ng/ml YM155 for 24h. The induction of apoptosis in the Mahlavu cells correlated well with the significant increase in cleaved caspase 3 and PARP (Fig. 4H). IHC and Western Blot further demonstrated the stimulation of γH2AX (pH2AX) expression in YM155-sensitive Mahlavu and HLE cells (Fig. 4H and 4I) compared to the YM155-resistant HuH7 and HepG2 cells (Fig. S3B), indicating acceleration of DNA damage. The ability of YM155 to inhibit the anchorage-independent cell growth, to induce cell cycle arrest, apoptosis and DNA damage of high survivin-expressing sensitive HCC cells suggests the therapeutic potential of YM155 on this molecular subtype of HCC cells.

To further decipher the molecular mechanisms involved in the induction of cell death by YM155 towards high survivin-expressing sensitive HCC cells, we performed comprehensive gene profiling analysis. Differentially expressed genes when Mahlavu cells were treated either with 10ng/ml YM155, 50nM survivin-specific siBIRC5, or the siRNA scramble control (Sigma), were identified. The knockdown of survivin by siRNA was validated by qRT-PCR (Fig. S4A) and silencing survivin decreased the sensitivity of Mahlavu cells to YM155 (Fig. S4B). Bioinformatics analysis of the differentially expressed genes identified showed that multiple cell-cycle, apoptotic and DNA-damage pathways were modulated (Fig. S4C and S4D) and these included the checkpoint regulatory-related genes CCNB1, CKS2, WEE1, API5 and GADD45A (Fig. S3E).

### YM155 is a promising anti-cancer agent for HCC cells with high survivin expression

**Figure 5:**
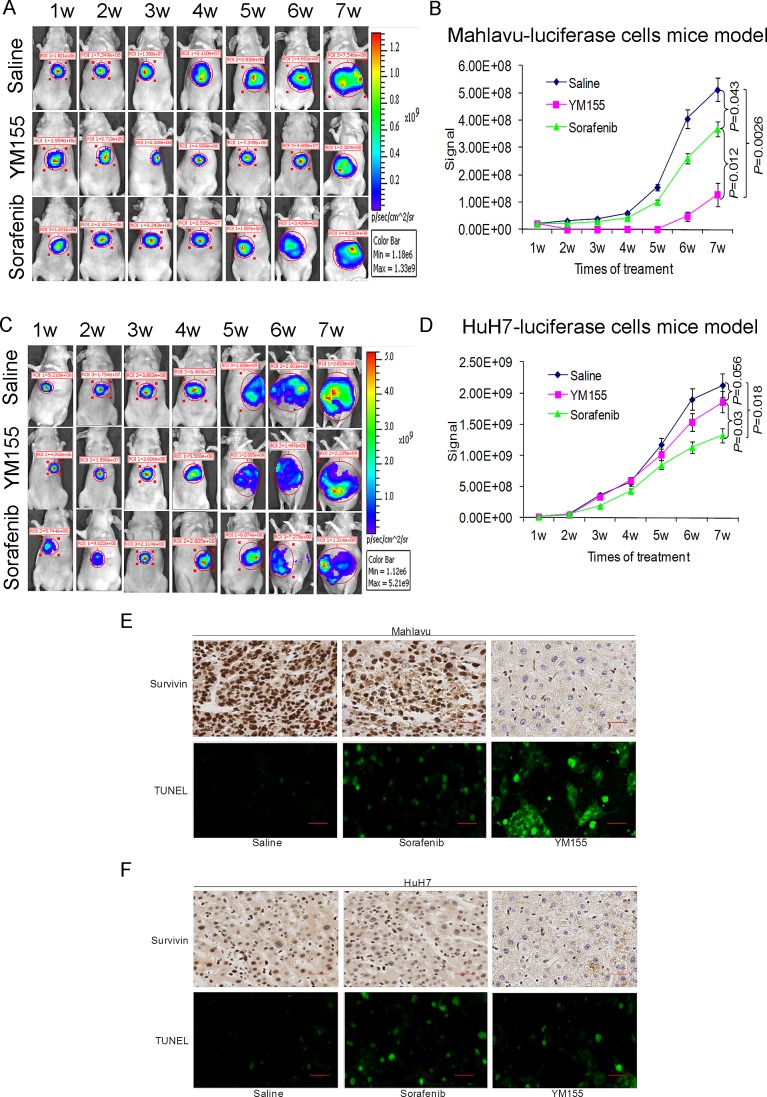
YM155 is a promising therapeutic agent for HCC cells with high survivin expression as demonstrated by the orthotopic liver tumor mouse model (A and C) Representative images obtained with the orthotopic mouse liver tumour xenograft model. (A) Mahlavu-luciferase cells (B) HuH7-luciferase cells. Weekly images following treatment with either saline, YM155 or sorafenib were shown. (B and D) Quantitative analysis of bioluminescence imaging signals obtained for all the mice following different treatments. (E and F) IHC analysis of survivin expression in (E) Mahlavu-induced tumor and (F) HuH7-induced tumor; the induction of tumor cell apoptosis (green TUNEL^+^ cells) was also studied by TUNEL staining in Mahlavu (E) and HuH7 (F) tumor.

We have stably transfected the pGL3 luciferase reporter vector into the Mahlavu and HuH7 cells. Nude mice orthotopically implanted with luciferase-expressing Mahlavu and HuH7 cells were treated either with YM155, sorafenib or saline for a total of 7 weeks. YM155 markedly suppressed the growth of the Mahlavu cells and the observed therapeutic effect, under these conditions, was statistically better than sorafenib (Fig. 5A and 5B). In comparison, the observed therapeutic effect of YM155 on HuH7, low surviving-expressing cells, was insignificant (Fig. 5C and 5D). TUNEL staining also confirmed that treatment with YM155 significantly increased apoptosis in tumors derived from the Mahlavu cells (Fig. 5E and 5F).

**Figure 6:**
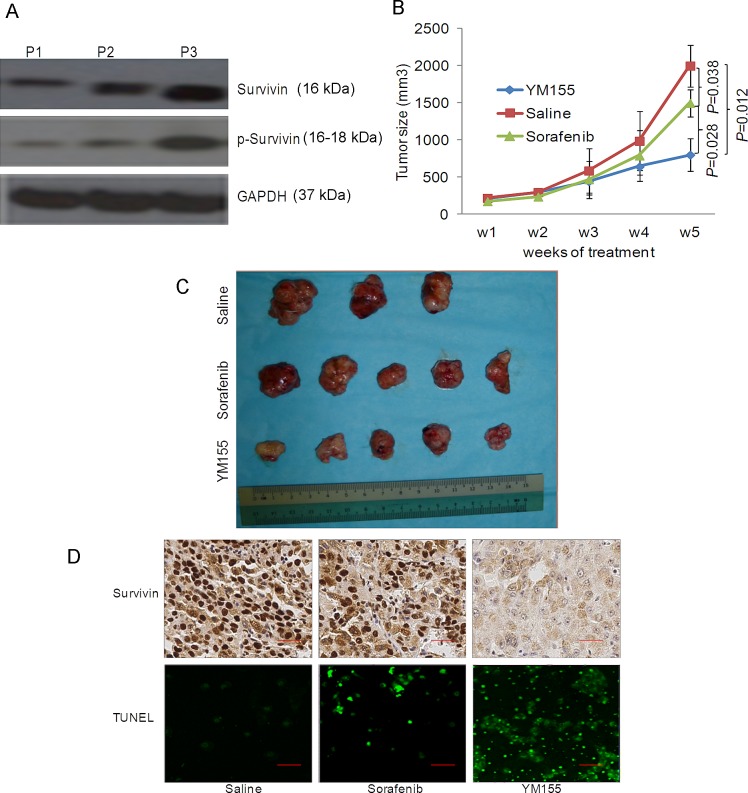
YM155 is a promising therapeutic agent for patient-derived HCC xenograft expressing high survivin (A) The expression of survivin and p-survivin in three patient-derived HCC tissue samples were studied by Western blot analysis. (B) Weekly quantitative analysis of the tumour volume of all mice receiving different treatments as shown. (C) Endpoint image of the tumor of the P3 patient-derived HCC tissue samples following treatment with saline, sorafenib and YM155. (D) Expression of survivin in the tumor tissues studied by IHC analysis and the induction of apoptosis was studied by TUNEL staining (green cells = TUNEL^+^ cells).

Previously, we have established several patient-derived HCC tumor xenograft lines [18]. One of these xenografts, tentatively designated as P3, expressed elevated level of survivin and p-survivin (Fig. 6A). P3 cells were employed in the orthotopic liver tumor mouse model for evaluating the anticancer efficacy of YM155. Although the growth of P3 was moderately inhibited by sorafenib, the antitumor effect of YM155 observed was significantly better under these conditions (Fig. 6B and 6C). Survivin expression was significantly inhibited in the YM155-treated mice and TUNEL assays performed on tumors isolated from the treatment groups further demonstrated a significantly increase of apoptotic cells in the YM155-treated group compared to treatment with sorafenib (Fig. 6D).

## DISCUSSION

While chemotherapy is one of the standard methods of treatment for many human cancers, systemic chemotherapy for HCC has been generally ineffective [19]. Currently, sorafenib is the first and only FDA-approved molecularly targeted therapy for advanced HCC. However, the clinical effectiveness of sorafenib has been unimpressive and the improvement in overall survival and time to progression is only approximately 3 months compared to placebo [6, 20]. Although novel molecularly targeted drugs such as sunitinib, erlotinib, linifanib, brivanib and everolimus are being developed, none of these drugs (insofar as information is available) has been dramatically effective [21]. One of the likely explanation is the existence of intra-tumor phenotypic heterogeneity which renders the accurate prediction of treatment response a major clinical challenge [22].

Survivin (BIRC5) is a member of the IAPs family that is overexpressed in most human cancers and is not expressed in normal tissues. In this study, we demonstrated that the expression and phosphorylation of survivin was highly heterogeneous in HCC. YM155 is a known inhibitor of survivin and has been the subject of several phase I and phase II clinical trials targeting diffuse large cell lymphoma, prostate cancer, melanoma, and NSCLC [12]. We demonstrated here that YM155-sensitive cells (Mahlavu and HLE) showed significantly elevated expression and phosphorylation of survivin while the YM155-resistant HCC cells (HuH7 and HepG2) gave relatively low survivin expression and phosphorylation.

Several clinical studies of YM155 against several human cancers have been reported but it has not been tested in HCC [12]. As a single agent, YM155 only produced modest activity in patients with refractory, advanced NSCLC [13]. The combination of YM155 with carboplatin and paclitaxel gave a favourable safety profile in advanced NSCLC but failed to produce an improvement in the response rate [23]. Currently, two trials involving YM155 and Rituximab against non-Hodgkin's lymphoma and YM155 and docetaxel against breast cancer are still active (http://clinicaltrials.gov/). Unfortunately, in all of these trials (Supplementary Table S2), the heterogeneity of survivin has not been considered in the protocol design and no patient selection strategy has been incorporated. This study clearly demonstrated that the level of survivin and p-survivin expression could serve as molecular predictive biomarkers to select potential YM155-responsive patients, in a move towards delivering precision medicine for HCC patients [24]. A >100-fold shifted in the sensitivity to YM155 was observed for the high survivin-expressing cells Mahlavu and HLE compared to the low survivin-expressing HuH7 and HepG2 cells.

Recently, Winter et al also reported the correlation of the expression of the solute carrier SLC35F2 with sensitivity to YM155 across a panel of cancer cell lines [25]. However, we have checked and observed that the expression of SLC35F2 in both our HCC dataset [22] and the reported HCC dataset of Roessler et al [17] was not differentially expressed between HCC and histologically adjacent normal clinical samples (Fig. S5A and S5B). Furthermore, we do not observe the heterogeneity of SLC35F2 expression associated with the HCC clinical tumor samples, indicating that SLC35F2 is unlikely to be the sole factor defining the heterogeneous sensitivity of HCC cells to YM155. The data presented here suggested that survivin and p-survivin expression could provide a comparatively better alternative for the stratification of HCC patients for treatment with YM155 in novel biomarker-driven therapeutic strategies.

## METHODS

### Cell viability

The cell viability was assessed by MTS [3-(4,5-dimethylthiazol-2-yl)-5-(3-carboxymethoxyphenyl)-2-(4-sulphophenyl)-2H-tetra zolium] assays using the CellTiter 96 AQueous One Solution Cell Proliferation Assay kit from Promega following the manufacturer's instructions. Each experiment was repeated three times.

### RNA extraction, microarray and RT-qPCR analysis

Total RNA was extracted using TRIzol reagent (Invitrogen) and the quality and quantity of isolated total RNA was assessed by the Agilent 2100 Bioanalyzer and NanoDrop ND-1000 Spectrophotometer (Agilent, Santa Clara, CA, USA). The microarray and *RT-qPCR analysis* were performed as described [15, 16]. Details are provided in Supplementary Materials and Methods.

### Western blotting, Immunohistochemistry (IHC) and immunofluorescence analysis

The procedure was performed as described [16] and the detail and antibodies were described in the Supplementary Materials and Methods.

### Flow-cytometry

The cell cycle and apoptosis was analysed by flow cytometry (FACSCanto II, BD Biosciences) using PI staining or Annexin V/7-AAD kits (BD Biosciences) according to the standard protocol.

### TUNEL assay

For labelling the nuclei of apoptotic cells, HCC cells were plated on glass coverslips in 24-well plates and fixed in 4% paraformaldehyde 24 hours post-YM155 treatment. TUNEL staining was performed using the DeadEnd fluorometric TUNEL system (Promega) according to the manufacturer's protocol. The number of TUNEL-positive cells was divided by the number of Hoechst 33342- stained cells to calculate the percentage of apoptotic nuclei.

### Clonogenicity assay

Cells were plated in 6-well plates and treated with YM155 (1ng/ml or 10 ng/ml) in culture medium. Upon the appearance of clones, the cells were fixed in methanol for 3 minutes and stained with a 0.01% crystal violet solution to assess colony formation. The number of macroscopically detectable colonies was registered. Treatments were performed in duplicate.

### Animal studies

All experiments on mice were approved by the SingHealth Institutional Animal Care and Use Committee (IACUC). Sorafenib was administered at levels effective on multiple tumor xenografts (30mg/kg po, daily). YM155 (10mg/kg) was administered via a 7-day continuous infusion by intraperitoneal injections and followed by observation for 7 days in 14-day treatment cycles. Tumor growth was monitored by bioluminescence imaging using the Xenogen IVIS Lumina system (Xenogen Corporation, Hopkinton, MA). Details of animal studies are provided in Supplementary Materials and Methods.

### Survival and statistical analysis

The experimental data are presented as the mean ± standard deviation (SD). All statistical analyses were performed using ANOVA or a two-tailed Student's *t* test by GraphPad Prism 5.0 (GraphPad Software, La Jolla, CA). The survival curves were created using the Kaplan-Meier method and statistically compared using a log-rank test. Differences were considered statistically significant when the P-values were less than 0.05.

## SUPPLEMENTARY MATERIAL, TABLES, FIGURES


