# Supplementary material for: The over-expression of survivin enhances the chemotherapeutic efficacy of YM155 in human hepatocellular carcinoma
Source: Oncotarget. 2015 Jan 21;6(8):5990–6000. doi: 10.18632/oncotarget.3337 (PMC4467416; doi:10.18632/oncotarget.3337)
Supplement: Supplementary file 1 [file oncotarget-06-5990-s001.pdf]

The over-expression of survivin enhances the chemotherapeutic efficacy of YM155 in human hepatocellular carcinoma

Supplementary Materials and Methods

Tissue specimens, cell lines and reagents

The collection of tumor and adjacent normal liver tissues from HCC patients were approved by our Institutional Review Board (IRB) and all tissues studied were provided by the Tissue Repository of the National Cancer Centre Singapore (NCCS) and Cancer Center, Sun Yat-Sen University. Written informed consent was obtained from all participating patients and all clinical and histopathological data provided to the researchers were rendered anonymous. Liver cancer cells were cultured in Dulbecco's modified Eagle's medium (HepG2, HuH7, PLC/PRF/5, HCCLM3, HLE, Mahlavu, SNU-449, SK-HEP-1), with 10% FBS and 100 units/mL of penicillin and 100 µg/mL of streptomycin (Invitrogen, Carlsbad, CA). All cell lines were maintained at 37°C in the presence of 5% CO_2. Sorafenib was from Bayer HealthCare Pharmaceuticals, Inc. and YM155 was synthesised by MedChemexpress Co. Ltd and Biochempartner Co. Ltd (Shanghai, China).

RNA extraction, microarray and Real-time quantitative RT-PCR (qRT-PCR) analysis

Total RNA from the tissue samples or cell lines was extracted using TRIzol reagent (Invitrogen). The quality and quantity of isolated total RNA were assessed using the Agilent 2100 Bioanalyzer and NanoDrop ND-1000 Spectrophotometer (Agilent, Santa

Clara, CA, USA). Microarray analysis was performed as described [22] using the GeneChip® Human Gene ST Arrays (Affymetrix, USA) according to the manufacturers' instructions. Post hybridization washes were performed on an Affymetrix GeneChip Fluidics Station 450. Arrays were scanned on an Affymetrix GeneChip Scanner 3000. Scanned arrays were normalized using GCRMA in Partek software (Partek Incorporated. St. Louis, MO). Array quality control was performed using Affymetrix® Expression Console™. Signal intensities were transformed to log2 base and imported to Partek Genomics Suite software (Partek Inc., St. Louis, MO) to conduct statistical analyses. The microarray data have been deposited in the European Bioinformatics Institutes of the European Molecular Biology Laboratory database (http://www.ebi.ac.uk/array express/) and are accessible through ArrayExpress public database with accession numbers E-MEXP-84 and E-TABM-292. The microarray data were further analysed using Ingenuity Pathway Analysis (IPA) Software. For mRNA detection by qRT-PCR, the total RNA was reversely transcribed by using SuperScript III First-Strand Synthesis System for RT-PCR (Invitrogen, CA). qPCR was performed using SsoFast™ EvaGreen® Supermix (Bio-Rad) with hypoxanthine phosphoribosyltransferase 1 (HPRT1) as an internal control as described previously, and fold changes were calculated via relative quantification ($2^{-\Delta Ct}$).

Western blotting

Protein was isolated using RIPA. Antibodies used for western blotting were: rabbit anti-survivin (#2808) or phospho-Survivin (Thr34) (D2E11) Rabbit mAb (#8888) (Cell Signaling, MA); mouse anti-PARP-1 and Caspase-3 (Santa Cruz, CA) and goat GAPDH (GenScript, NJ).

Immunohistochemistry (IHC)

The IHC was performed as described previously. Primary antibodies used included the rabbit anti-survivin (#2808) (Cell Signaling, MA) (1:100), or an isotype-matched IgG as a negative control. Intensity of staining was evaluated based on a scale of 0 to 4 according to the percentage of positive tumours (0, negative control; +, 0%-10%; ++, 10%-25%; +++, 25%-50% and ++++, >50%).

Immunofluorescence analysis

HCC cells were seeded in the BD Falcon™ 8-well CultureSlide and treated with YM155 (10 ng/ml) for 24 hours. The treated cells were fixed and incubated with primary antibodies against p-H2AX and then incubated with Alexa Fluor® 488 goat anti-rabbit IgG (Invitrogen). Slides were counterstained with Hoechst 33342 and imaged using a confocal laser-scanning microscope (Carl Zeiss).

Animal studies

All experiments were approved by the Institutional Animal Care and Use Committee of SingHealth. All mice studies were done in the Animal Unit with AAALAC accreditation in National Cancer Center Singapore. Mahlavu- and HuH7-luciferase expressing cells were firstly subcutaneously injected into 5- to 6-week-old athymic mice (2 mice per group, BioLASCO Taiwan Co, Ltd) to generate tumor. The tumor-bearing mice were anesthetized with Hypnorm/Midazolam and a small piece of tumor was harvested and orthotopically transplanted to the liver of the recipient mice as described previously [18]

to establish the orthotopic mouse liver tumor xenograft model. One week after implantation, the mice were imaged and grouped. Mice with similar signals were grouped (10 mice/group) and treated either with YM155, sorafenib or saline for 7 weeks. Sorafenib was administered at the effective dose of 30 mg/kg/dose, p.o. Mice were treated with YM155 for 7 days with a continuous intraperitoneal injection of 10 mg/kg, followed by a 7-day rest period. Each cycle was repeated every 14 days for a total of 4 cycles of treatment. Tumor growth was monitored every week by bioluminescence imaging using the Xenogen IVIS Lumina system (Xenogen Corporation, Hopkinton, MA).

Supplementary Table

Table S1: Relationship between survivin expression and clinicopathological parameters of HCC

Patients' characteristics	Number of cases	Survivin expression		p-value
		Low	High	
**Age (years)**				
≤60	36	18	18	
>60	40	21	19	0.828
**Sex**				
M	66	36	30	
F	10	3	7	0.148
**Tumor size (cm)**				
≤3	23	14	9	
>3	53	25	28	0.272
**AJCC tumor staging**				
I	46	27	19	
II	21	8	13	
III A	9	4	5	0.267
**AFP (ng)**				
≤20	38	26	12	
>20	38	13	25	**0.003**
**Tumor venous infiltration**				
VI	28	12	16	
NI	48	27	21	0.26
**Child's grade (A) (B)**				
A	56	33	23	
B	20	6	14	**0.026**
**No. of tumor nodules**				
1	67	32	35	
M	9	7	2	0.091
**Cirrhosis**				
Y	36	16	20	
N	35	21	14	0.19
**Recurrence (Months)**				
≤24	36	14	22	
>24	35	23	12	**0.024**

Table S2: The current clinical trial of YM155 searching on https://clinicaltrials.gov.

Rank	Status	Study
1	Completed	A Clinical Pharmacological Study of YM155 After Intravenous Infusion in Patients With Advanced Cancer<br>Condition: Cancer<br>Intervention: Drug: YM155
2	Completed	An Extension Study Administering YM155 to Subjects Previously Enrolled in Another Protocol Administering YM155<br>Conditions: Prostate Cancer; Melanoma; Non-Hodgkin's Lymphoma<br>Intervention: Drug: YM155
3	Completed	A Study of YM155 Plus Docetaxel in Subjects With Stage III (Unresectable) or Stage IV Melanoma<br>Condition: Melanoma<br>Interventions: Drug: YM155; Drug: Docetaxel
4	Active, not recruiting	A Study of YM155 Plus Rituximab in Subjects With Non-Hodgkin's Lymphoma Who Have Received Prior Treatment<br>Condition: Non-Hodgkin's Lymphoma<br>Interventions: Drug: YM155; Rituximab
5	Completed	A Study of YM155 Plus Docetaxel as First-Line Treatment in Subjects With HER2 Negative Metastatic Breast Cancer<br>Condition: Breast Cancer<br>Interventions: Drug: YM155; Drug: Docetaxel
6	Completed Has Results	A Phase I/II Study of Paclitaxel, Carboplatin and YM155 (Survivin Suppressor) in Subjects With Solid Tumors (Phase I) and Advanced Non-Small Cell Lung Carcinoma (Phase II)<br>Conditions: NSCLC; Solid Tumors<br>Interventions: Drug: YM155; Drug: Carboplatin; Drug: Paclitaxel
7	Terminated	Study of YM155 in Refractory Diffuse Large B-Cell Lymphoma (DLBCL) Subjects<br>Conditions: Lymphoma; Lymphoma, B-Cell Refractory<br>Intervention: Drug: YM155
8	Completed	An Open-Label Study of YM155 + Docetaxel in Subjects With Advanced Hormone Refractory Prostate Cancer and Other Solid Tumors<br>Conditions: Prostate Cancer; Tumors<br>Interventions: Drug: YM 155; Drug: Docetaxel; Drug: Prednisone
9	Completed	A Study for the Treatment of Unresectable Stage III or Metastatic Stage IV

		Melanoma<br>Condition: Melanoma<br>Intervention: Drug: YM155
10	Completed	A Study for the Treatment of Hormone Refractory Prostate Cancer (HRPC) in Patients Previously Treated With Chemotherapy<br>Conditions: Prostate Cancer; Cancer of Prostate; Prostatic Cancer; [illegible]<br>Intervention: Drug: YM155
11	Completed	LUCY: A Study for the Treatment of Non-Small Cell Lung Cancer (NSCLC) in Patients Previously Treated With Chemotherapy<br>Conditions: Lung Cancer<br>Intervention: Drug: YM155

Supplementary Figures:

A

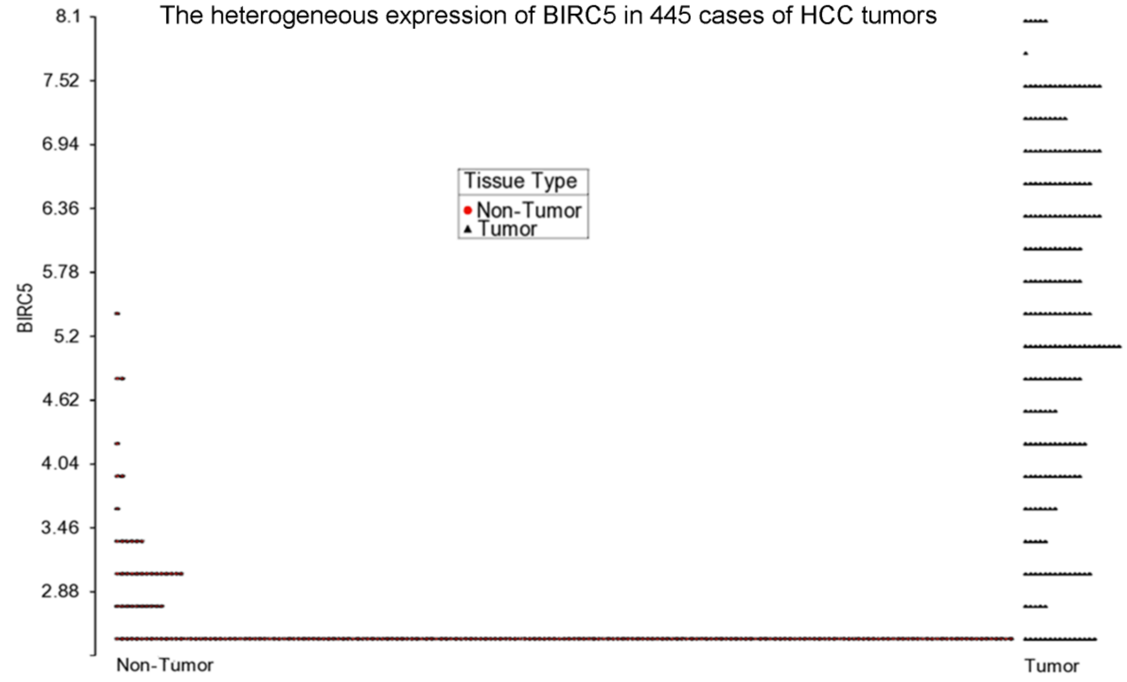

B

TMA102														
	1	2	3	4	5	6	7	8	9	10	11	12	13	14
A	311				312				321				324	
B			327				329				330			
C	336				339				361				369	
D			373				383				400			
E	409				418				440				449	
F			452				495				508			
G	509				525				528				543	
H			545				548				554			
I	592				X	X	598				X	X	X	X

Fig. S1: The expression of survivin (BIRC5) in the HCC samples reported by Roessler et al. (A) Expression of survivin was shown by dot plot analysis after searching the Gene Expression database available in GEO (gse14520_raw). (B) The ID number of HCC samples employed for IHC staining in a tissue array to validate the expression of survivin in an independent panel of HCC tumor tissues.

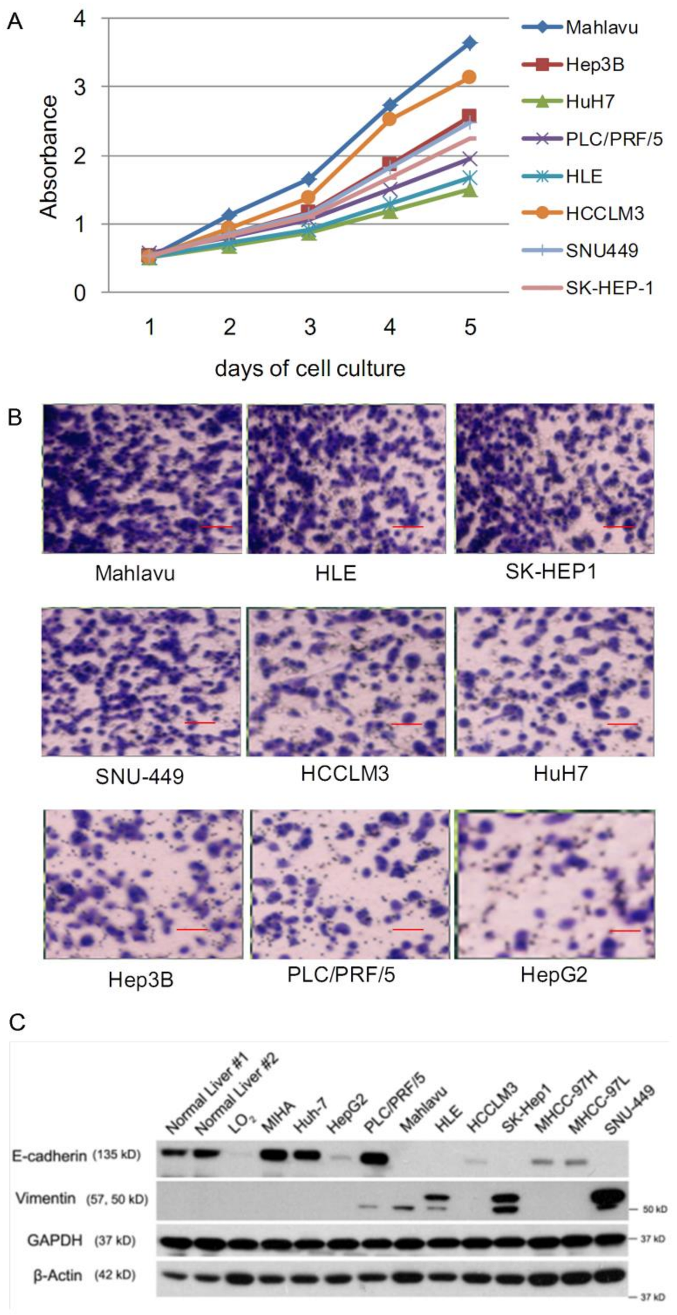

Fig.S2: The cell growth rates (A), invasiveness (B), and EMT markers (Vimentin and E-cadherin) expression (C) were measured in a panel of liver cancer cells with different levels of survivin.

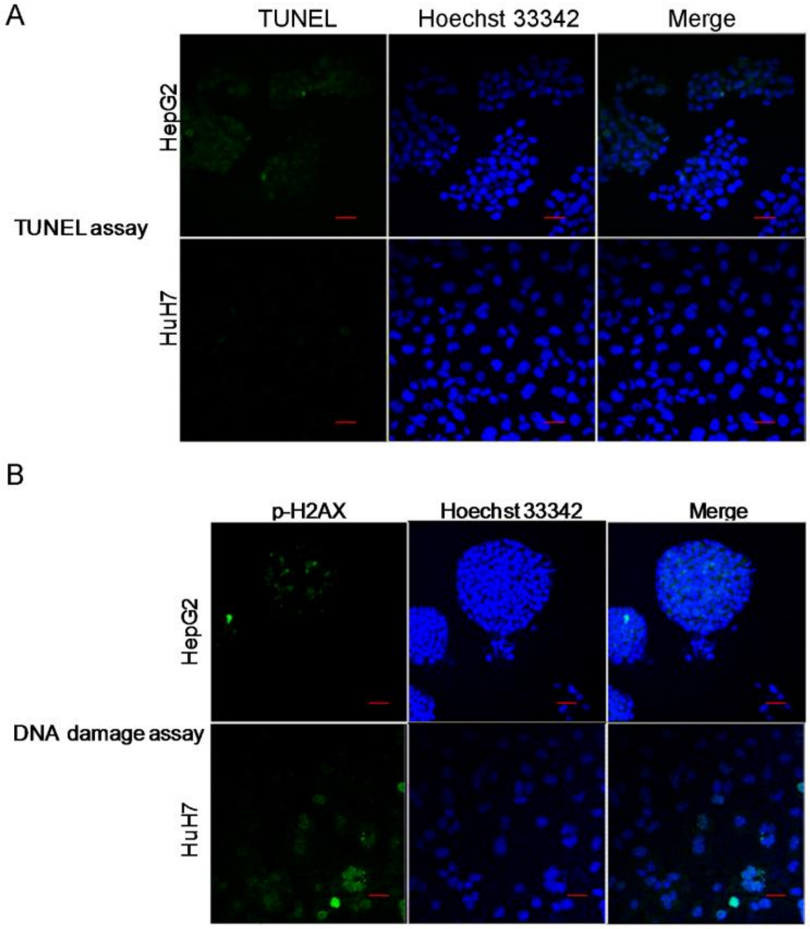

Fig. S3: Effects of YM155 on the apoptosis and DNA damage of HuH7 and HepG2 cells. (A) The represent images of TUNEL staining. There were few TUNEL$^+$ cells observed by confocal microscopy analysis in both HuH7 and HepG2 cells treated with 10ng/ml YM155. The green signal represents staining for TUNEL$^+$ cells. Nuclear DNA was detected by staining with Hoechst 33342. (B) The represent images of DNA damage marker p-H2AX staining. The HuH7 and HepG2 cells were treated with 10ng/ml YM155 and stained for p-H2AX and Hoechst 33342 before being analysed by confocal microscopy. The green signal represents staining for p-H2AX. Nuclear DNA was detected by staining with Hoechst 33342.

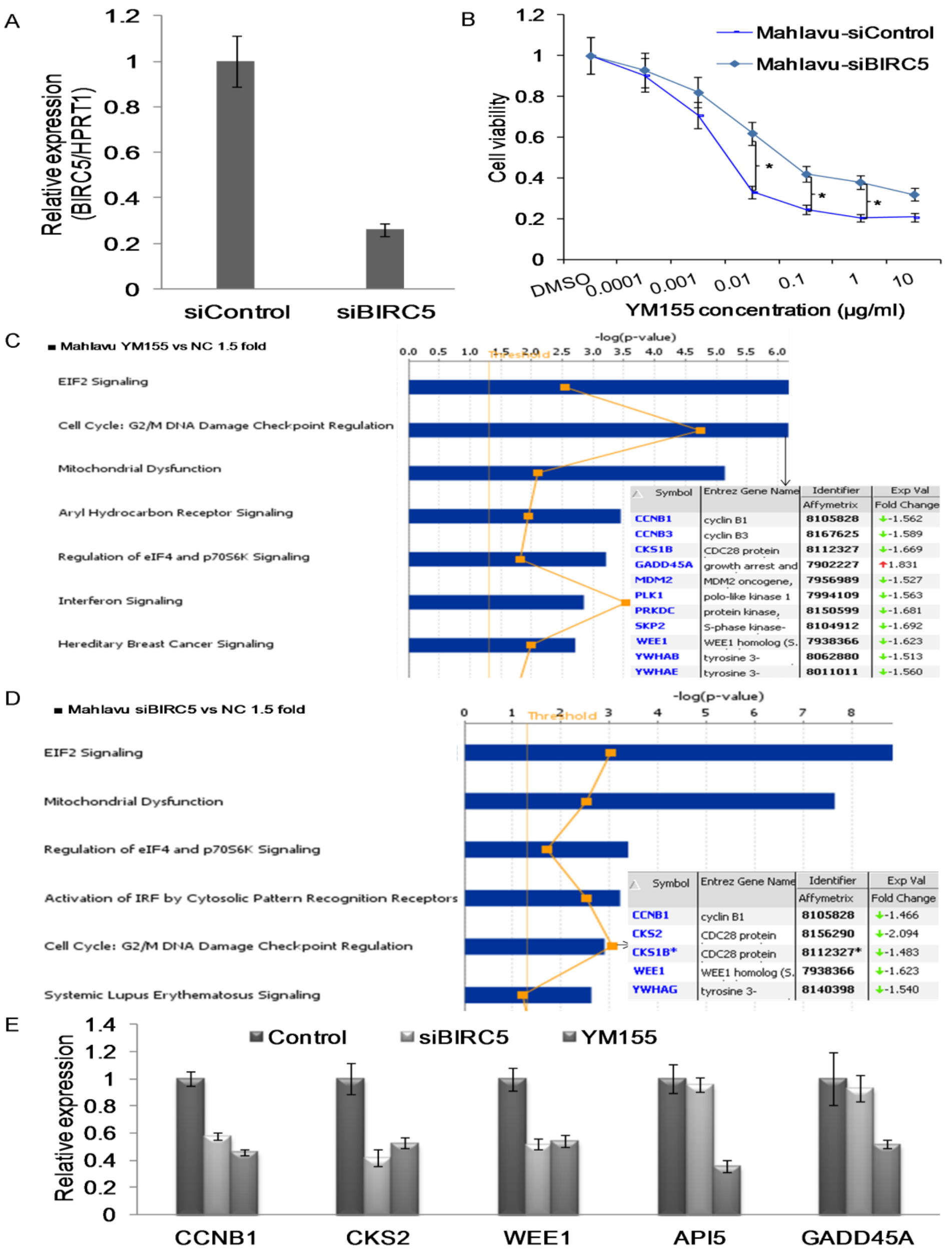

Fig. S4: Knockdown survivin decreased the cell sensitivity to YM155 and the possible underlying molecular mechanisms involved in the induction of cell death by YM155. (A) The knockdown of survivin by siRNA transfection using qRT-PCR analysis. (C and D) Ingenuity Pathway Analysis using the differentially expressed genes identified using microarrays. (C) 10ng/ml YM155-treated Mahlavu cells and control Mahlavu cells, or (D) Survivin siRNA (50nM)-treated Mahlavu cells and control Mahlavu cells using 1.5-fold in differential expression to generate the gene list to construct the networks. (E) Effects of YM155 and survivin knockdown on the cell cycle G2/M DNA damage checkpoint regulatory-related genes (CCNB1, CKS2, WEE1, API5 and GADD45A) using real time RT-qPCR analysis.

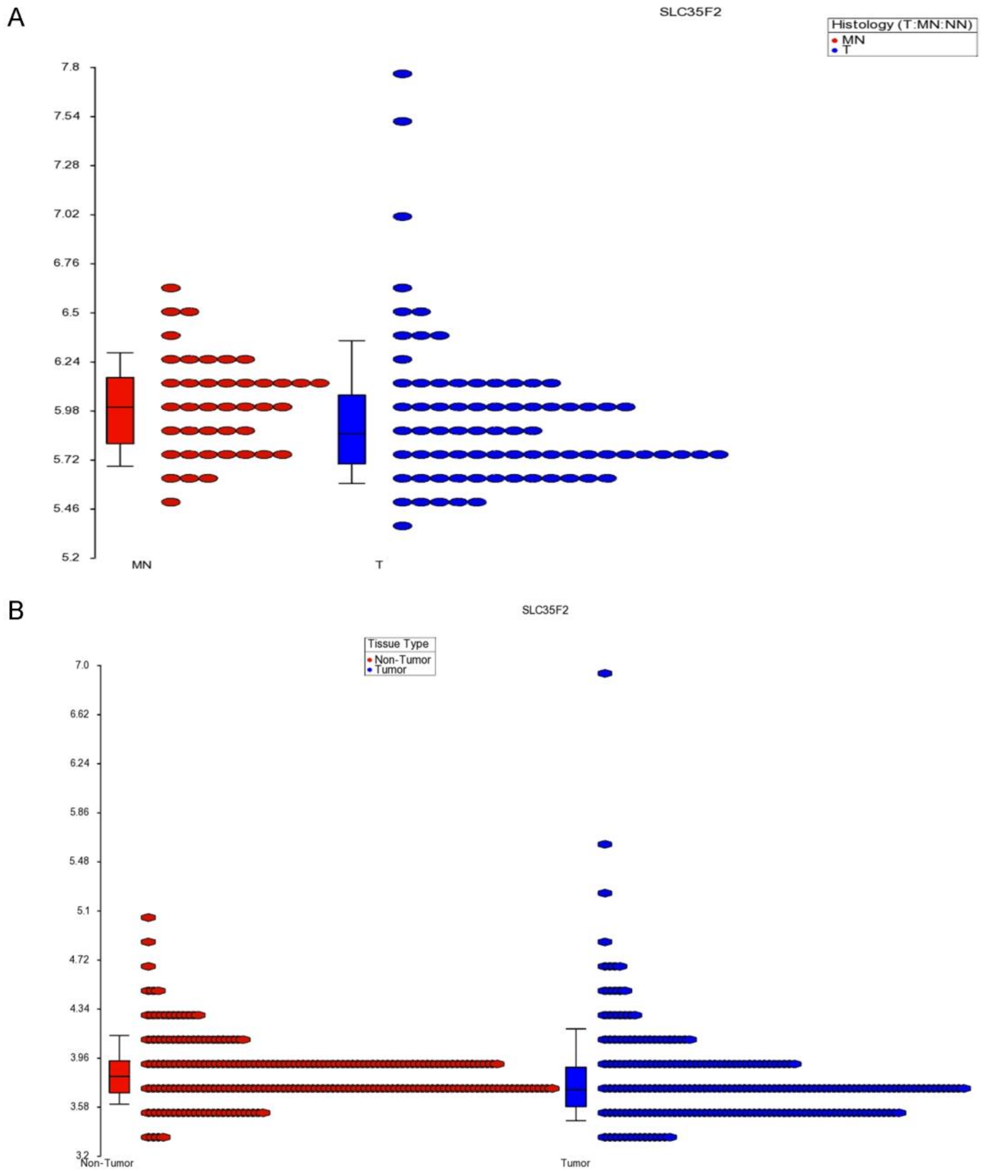

Fig. S5: The expression of SLC35F2 in our HCC dataset (A) and in the published HCC dataset of Roessler et al [23] (B).
